# Tear glucose is associated with the presence and severity of diabetic retinopathy

**DOI:** 10.1186/s40942-025-00636-x

**Published:** 2025-02-06

**Authors:** Ningyao Cao, Lili Feng, Wei Xu, Fanglin He, Caiwen Xiao, Yan Liu, Weirong Xu, Jingjing Cui, Yuqian Guo, Lianqing Yao, Wenwen Xia, Fei Chen, Yong Li, Chuandi Zhou, Xiaofang Xu

**Affiliations:** 1 Department of Ophthalmology, Ninth People’s Hospital, Shanghai Jiao Tong University School of Medicine, No. 639 Zhizaoju Road, Shanghai, 200011 China; 2 Shanghai Key Laboratory of Orbital Diseases and Ocular Oncology, Shanghai, China; 3 Department of Cooperation and Exchange, Shanghai General Hospital, Shanghai Jiao Tong University School of Medicine, Shanghai, China; 4 Department of Anesthesiology and Operating Room, The Ninth People’s Hospital of Shanghai, Jiao Tong University School of Medicine, No. 639 Zhizaoju Road, Shanghai, 200011 China; 5 Department of General Medicine, Shanghai General Hospital, Shanghai Jiao Tong University School of Medicine, Shanghai, China; 6 Department of Ophthalmology & Otolaryngology, Qiaojia County Traditional Chinese Medicine Hospital, Zhaotong, Yunnan China; 7 Department of Pathology, Ninth People’s Hospital, Shanghai Jiao Tong University School of Medicine, Shanghai, China; 8 Department of Ophthalmology, The Calmette Affiliated Hospital of Kunming Medical University, No. 1228 Beijing Road, Panlong District, Kunming, 650021 Yunnan China; 9 Innovative Research Team of High-Level Local Universities in Shanghai, Shanghai, China

**Keywords:** Diabetic retinopathy, Tear glucose, Diabetes, Cross-sectional study

## Abstract

**Purpose:**

To examine the association between tear glucose (TG) and the presence and severity of diabetic retinopathy (DR) in patients with type 2 diabetes mellitus (T2DM).

**Methods:**

A cross-sectional study. TG was examined by rapid qualitative test strip in 160 patients. The severity of DR was graded as mild DR and severe DR. The presence and severity of DR were compared between patients with positive and negative TG. The association of TG with the presence and the severity of DR was estimated by multivariable regression analysis and spearman’s rank correlation test, respectively. The performance of TG to detect DR was evaluated by the receiver operating characteristic (ROC) curve.

**Results:**

In this study, 160 patients were included, with a median age of 64.0 years, and 88 (55.0%) patients were males. A total of 91 (56.9%) patients had positive TG, and 69 (43.1%) patients had negative TG. In TG-positive group, 41 (45.1%) patients were diagnosed with DR, among them, 8 (19.5%) patients had mild DR, and 33 (80.5%) patients were afflicted with severe DR. Multivariable logistic regression indicated that the presence of DR positively correlated with the presence of positive TG (odds ratio [OR], 3.62; 95% confidence interval [CI], 1.56–8.40; p < 0.01), longer duration of diabetes (OR, 1.11; 95% CI 1.06–1.17; p < 0.01) and higher HbA1c (OR, 1.25; 95% CI 1.01–1.54; p = 0.03). Moreover, Spearman’s correlation analysis suggested that the grading of TG increased with the severity of DR (r_s_ = 0.28, p < 0.01). The area under the curve (AUC) of the model integrating TG, the duration of diabetes and HbA1c was 0.76 (95% CI 0.69–0.84), indicating a fair discriminative ability of DR.

**Conclusion:**

TG level was associated with the presence and the severity of DR. TG might be an easy-to-use, non-invasive parameter to the screening and monitoring of DR among patients with diabetes.

**Supplementary Information:**

The online version contains supplementary material available at 10.1186/s40942-025-00636-x.

## Introduction

In the twenty-first century, diabetes mellitus (DM) is on the rise globally [1]. The number of people with DM worldwide had reached 828 million in 2022, with a substantial proportion of them living in developing countries [2]. Standardized glycemic control is crucial to prevent the development of DM and its complications [3], such as diabetic retinopathy (DR), nephropathy, neuropathy, coronary heart disease and et al [4]. One of the most prevalent diabetic microvascular complication is DR [5]. DR is a primary cause of vision impairment and blindness in working-age population globally [6]. Facing the huge burden of DR, accurate assessment and early diagnosis of DR are critical to improving visual outcomes.

Current standards of glycemic control require self-monitoring of glucose several times daily [7]. Finger-prick glucose testing is the most prevalent self-measurement of blood glucose (BG) for the majority of patients [8]. However, this method is invasive [9] and inconvenient, for it causes pain, loss of finger sensitivity and scar formation [10], which compromises the compliance of patients. Commercialized continuous glucose monitoring devices are minimally invasive [10], nevertheless, they are costly for long-term using.

In recent years, many alternative ways for BG monitoring have been developed [11]. Tears, readily available and non-invasive body fluids containing glucose, have become an interesting matrix for many decades [12,13]. Evidence is booming to prove a solid correlation between tear glucose (TG) and BG [14,15]. Compared to normal individuals, patients with diabetes have higher TG level [16]. Therefore, TG testing may offer a noninvasive and effective option for self-management in patients with diabetes.

Poor glycemic control aggravates DR [17]. For TG is positively correlated with BG [14], and TG might could have reflected the presence and severity of DR. However, to our knowledge, the association of TG and DR has not been explored.

This cross-sectional study measured TG levels of patients with type 2 diabetes, and assessed their retinal status, with the purpose to examine the association between TG and DR. Provided TG is correlated with the presence and severity of DR, it might have become an effective, noninvasive and convenient biomarker for self-monitoring of BG, DR as well as other micro- and macro-vascular complications of diabetes.

## Methods

### Data collection

The purpose of this research was explained to each participant. The whole participant voluntarily joined in our study and were not compensated for their involvement. Written informed consent was obtained from all patients. This study adhered to the tenets of the Declaration of Helsinki. Our study was approved by the institutional review board of the Shanghai Ninth People's Hospital, Shanghai Jiao Tong University School of Medicine.

A total of 170 patients with type 2 diabetes were recruited from the Department of Ophthalmology, Ninth People's Hospital, Shanghai Jiao Tong University School of Medicine between June 2018 and May 2021. In final analysis, 10 patients with serious diseases of visual damage other than DR, such as primary glaucoma, neovascular age-related macular degeneration (nAMD), and one or both eyes without light perception, were excluded. Finally, 160 individuals were eligible for this study.

A comprehensive ophthalmic evaluation of DR consisted of slit-lamp examinations, optical coherence tomography (OCT) (Germany) and color fundus photography (CFP) (Germany) through a dilated pupil. The Early Treatment Diabetic Retinopathy Study (ETDRS) [18] depicted the retinal lesions, including intraretinal microvascular, microaneurysms of retina, soft or hard exudates, blot hemorrhages, venous beading, abnormalities, proliferative membrane, neovascularization of retina, photocoagulation scars from laser scatter, preretinal or vitreous hemorrhage and tractional retinal elevation [18]. DR was defined as the occurrence of any above-mentioned lesion. The ETDRS grading standards, mild-moderate non-proliferative DR (NPDR) (ETDRS level 20–47), severe NPDR (ETDRS level 53) and proliferative DR (PDR) (ETDRS level ≥ 60), were used to define the severity of DR [18]. For patients with bilateral disease, the worse eye was selected for analysis. The patients with DR were divided into mild DR and severe DR, in which, mild DR included mild and moderate NPDR, and severe DR consisted of severe NPDR and PDR. The retinopathy was assessed by two vitreoretinal specialists (X.X., C.Z.) every patient. In the first-round, the consistency of assessment between two specialists was 96.88%. The third specialist (Y.L.) was invited to deliberate on inconsistency.

We collected the data including baseline demographics and clinical characteristics. The demographics included age and gender. The clinical characteristics included the duration of diabetes, the levels (grade 0–4; negative TG: 0, positive TG: 1–4) of TG obtained by rapid qualitative test strip in the outpatient department. Laboratory testing of blood samples, including fasting plasma glucose (FPG) and glycated hemoglobin (HbA1c) within one month were also collected.

Tear fluid was collected using rapid TG qualitative test strip. In brief, the tear sample collection region of the strip was placed into the lateral canthus for 30–60 s to allow for tear absorption. Tear samples were carefully collected in avoid to contact the conjunctiva. The collected tear samples went up through the pH buffer region to neutralize the sample and then arrived at the glucose test region (Figure S1). The TG concentration was analyzed by glucose oxidase coupled with Trinder’s reaction.

Comparing the color of tear samples with the standard color blocks. If the tear sample of the color development was equal to or lighter than S1, the result was grade 0 and TG concentration ≤ 0.15 mmol/L; If the tear sample of the color development was darker than S1 or equal to S2, the result was grade 1 and TG concentration between 0.15 mmol/L and ≤ 0.30 mmol/L; If the tear sample of the color development was darker than S2 or equal to S3, the result was grade 2 and TG concentration between 0.30 mmol/L and ≤ 0.60 mmol/L; If the tear sample of the color development was darker than S3 or equal to S4, the result was grade 3 and TG concentration between 0.60 mmol/L and ≤ 1.20 mmol/L; If the tear sample of the color development was darker than S4, the result was grade 4 and TG concentration > 1.20 mmol/L (Figure S2).

## Statistical analysis

All analyses were performed with SPSS software (version 27.0). Respectively, the frequency (percentage) and median (inter-quartile range [IQR]) were given for the description of categorical and continuous variables. The baseline demographics and clinical characteristics were compared between the patients with positive and negative TG by using either the Mann–Whitney U test (continuous factors) or the chi-square test (categorical factors). Multivariable logistic regression model was constructed to assess the correlation between TG and DR. Chi-square test was applied to identify the association between the TG and the presence of DR with Bonferroni's correction used for subsequent multiple comparisons. Spearman’s rank correlation test was used to identify the correlation between TG level and the severity of DR, and a strong correlation was defined as a correlation coefficient greater than 0.4 and a p value less than 0.05. The sensitivity, specificity, Jouden index, odds ratio, receiver operating characteristic (ROC) and area under the curve (AUC) were used to evaluate the discriminative ability of TG to detect DR. The odds ratios (ORs) with 95% confidence intervals (CIs) were figured out. A p value < 0.05 was considered statistically significant.

## Results

### Descriptive statistics

Of the 160 patients recruited in this study, 88 (55.0%) were male and 72 (45.0%) were female. The median age was 64.0 years, ranging from 27.0 to 89.0 years (IQR, 56.0–70.0 years). The median duration of diabetes was 10.0 years (IOR, 5.0–16.0 years). In our cohort, 91 (56.9%) patients exhibited positive-TG and 69 (43.1%) patients had negative TG. The baseline demographics and clinical characteristics were compared between the patients with positive and negative TG. Significant difference was noted regarding FPG (7.1 vs. 6.6 mmol/L, p = 0.02). Marginally significant difference was noted in HbA1c (7.2 vs 6.8%, p = 0.05). No remarkable differences were observed in gender (p = 0.76), age (p = 0.06), and the duration of diabetes (p = 0.66) between the two groups (Table 1).

**Table 1:** Baseline demographics and clinical characteristics

Variables	Total (n = 160)	Tear glucose ( +) (n = 91)	Tear glucose (-) (n = 69)	P
Sex				0.76
Male	88(55.0)	51(56.0)	37(53.6)	
Female	72(45.0)	40(44.0)	32(46.4)	
Age (years)	64.0(56.0–70.0)	62.0(55.0–68.0)	66.0(57.0–72.0)	0.06
Duration of diabetes(years)	10.0(5.0–16.0)	10.0(5.0–18.0)	10.0(3.5–15.0)	0.66
HbA1c(%)	7.1(6.4–8.2)	7.2(6.4–8.7)	6.8(6.2–7.4)	0.05*
FPG(mmol/L)	6.8(6.3–8.0)	7.1(6.3–8.6)	6.6(6.2–7.2)	0.02*

Data are presented as median (interquartile range) or n (%)

HbA1c: glycosylated hemoglobin; FPG: fasting plasma glucose

^*^Statistically significant

### The association between TG and DR

In this cohort, 53 (33.1%) patients were diagnosed with DR. In TG-positive group, 41 (45.1%) patients were diagnosed as DR, which was significantly higher than that of TG-negative group (17.4%, p < 0.01). Univariable logistic regression indicated that younger age (OR, 0.97; 95% CI 0.94–1.00; p = 0.03), longer duration of diabetes (OR, 1.08; 95% CI 1.04–1.13; p < 0.01), the presence of positive TG (OR, 3.90; 95% CI 1.85–8.22; p < 0.01), higher HbA1c (OR, 1.27; 95% CI 1.05–1.54; p = 0.01) and FPG (OR, 1.35; 95% CI 1.14–1.59; p < 0.01) were the potential correlates of DR. Furthermore, multivariable logistic regression showed that younger age (OR, 0.94; 95% CI 0.91–0.98; p < 0.01), longer duration of diabetes (OR, 1.11; 95% CI 1.06–1.17; p < 0.01), the presence of positive TG (OR, 3.62; 95% CI 1.56–8.40; p < 0.01) and higher HbA1c (OR, 1.25; 95% CI 1.01–1.54; p = 0.03) were independently associated with the presence of DR (Table 2).

**Table 2:** Logistic regression analysis of diabetic retinopathy

Variables	DR ( +) (n = 53)	DR (-) (n = 107)	Univariable Logistic Regression			Multivariable Logistic Regression		
			OR	95%CI	p	OR	95%CI	p
Sex			0.81	0.42–1.57	0.53			
Male	31(58.5)	57(53.3)						
Female	22(41.5)	50(46.7)						
Age (years)	61.0(54.0–68.0)	65.0(58.0–71.5)	0.97	0.94–1.00	0.03*	0.94	0.91–0.98	< 0.01*
Duration of diabetes(years)	15.0(10.0–20.0)	8.0(3.0–14.0)	1.08	1.04–1.13	< 0.01*	1.11	1.06–1.17	< 0.01*
Tear glucose			3.90	1.85–8.22	< 0.01*	3.62	1.56–8.40	< 0.01*
Positive	41(77.4)	50(46.7)						
Negative	12(22.6)	57(53.3)						
HbA1c(%)	7.8(6.4–8.9)	6.8(6.3–7.8)	1.27	1.05–1.54	0.01*	1.25	1.01–1.54	0.03*
FPG(mmol/L)	7.2(6.4–9.2)	6.6(6.3–7.6)	1.35	1.14–1.59	< 0.01*			

Data are presented as median (interquartile range) or n (%)

CI, Confidence interval; OR, Odds ratio

HbA1c: glycosylated hemoglobin; FPG: fasting plasma glucose; DR: diabetic retinopathy

^*^Statistically significant

### The association between the level of TG and the severity of DR

Spearman’s correlation analysis suggested that TG level was positively correlated with the severity of DR (r_s_ = 0.28, p < 0.01) (Table 3). Among patients with positive TG, 8 (8.8%) had mild DR and 33 (36.3%) had severe DR. The rate of positive TG in patients with severe DR is 82.5%, which was significantly higher than that of patients with no DR (46.7%, p < 0.01). A notable trend was indicated that a higher level of TG with increasing severity of DR (Table 4).

**Table 3:** The association between tear glucose grading and the severity of diabetic retinopathy

Variables	No DR (n = 107)	DR( +) (n = 53)		p
		Mild DR (n = 13)	Severe DR (n = 40)	
Tear glucose grading				< 0.01*
Grade 0	57(53.3)	5(38.5)	7(17.5)	
Grade 1	8(7.5)	2(15.4)	6(15.0)	
Grade 2	11(10.3)	1(7.7)	10 (25.0)	
Grade 3	14(13.1)	1(7.7)	3 (7.5)	
Grade 4	17(15.9)	4(30.8)	14 (35.0)	

Data are presented as n (%)

DR: diabetic retinopathy. NPDR: non-proliferative diabetic retinopathy

Mild DR: mild and moderate non-proliferative diabetic retinopathy; Severe DR: severe non-proliferative diabetic retinopathy and proliferative diabetic retinopathy

^*^Statistically significant

**Table 4:** The distribution of different status of tear glucose stratified by severity of diabetic retinopathy

Tear glucose	No DR (n = 107)	Mild DR (n = 13)	Severe DR (n = 40)	p	p^†^	p^‡^
Positive	50(46.7)	8(61.5)	33(82.5)	< 0.01^*^	0.31	< 0.01^*^
Negative	57(53.3)	5(38.5)	7(17.5)			

Data are presented as n (%)

DR: diabetic retinopathy; TG: Tear glucose; Mild DR: mild and moderate non-proliferative diabetic retinopathy; Severe DR: severe non-proliferative diabetic retinopathy and proliferative diabetic retinopathy

*Statistically significant; †Mild DR vs No DR; ‡Severe DR vs No DR

Significant differences were calculated by Bonferroni’s post hoc comparison test

### Discriminative value of TG in DR

**Fig. 1:**
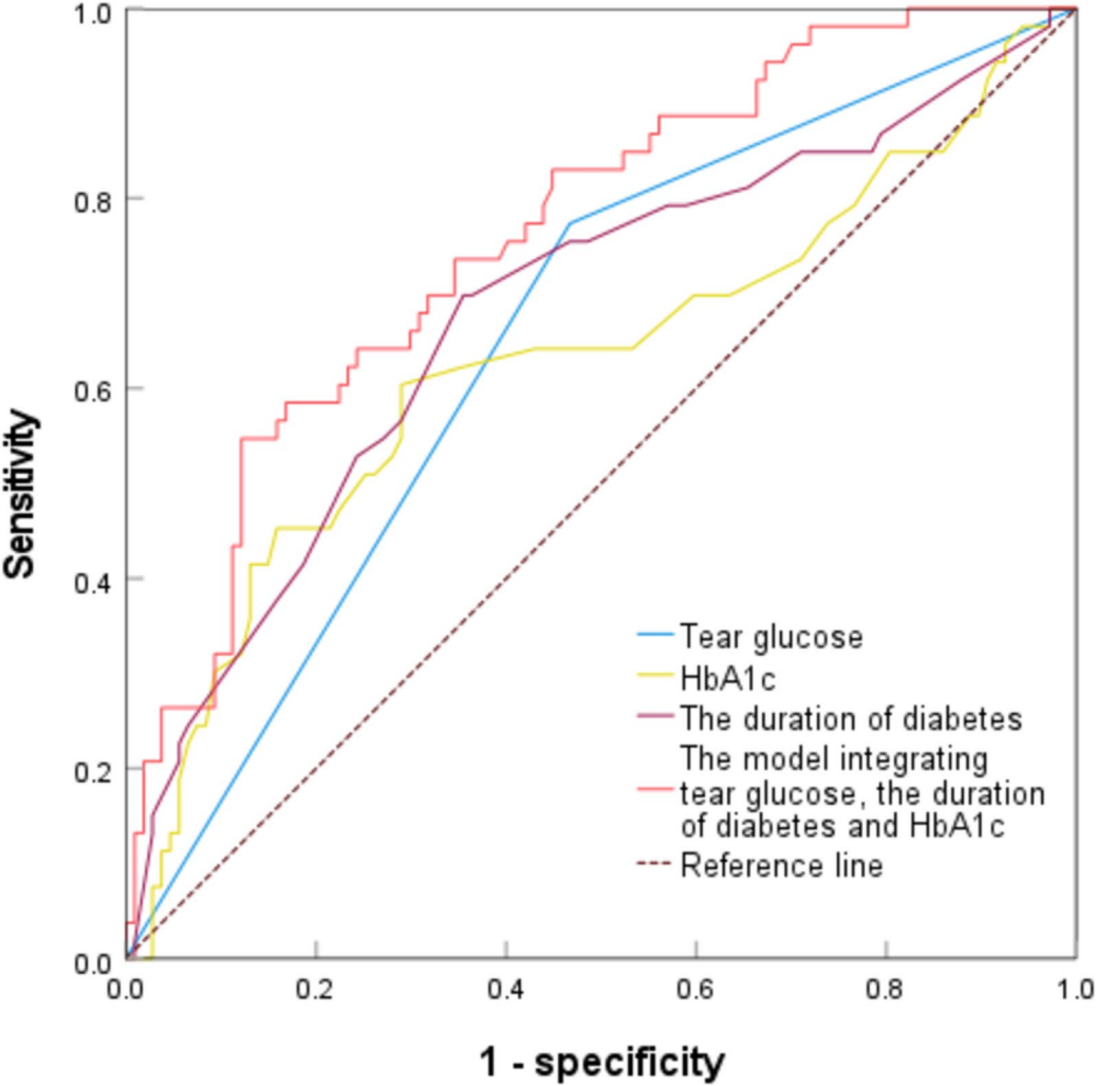
ROC curves comparing tear glucose, the duration of diabetes, HbA1c and the model integrating tear glucose, the duration of diabetes, HbA1c for detection of diabetic retinopathy

The ROC curves were displayed in Fig. 1, in which, the AUC of the model integrating HbA1c, TG, and the duration of diabetes was 0.76 (95% CI 0.69–0.84), indicating a fair discriminative ability to identify DR, which was significantly higher than the AUCs of each element alone (p < 0.01) (Table 5).

**Table 5:** Receiver operating characteristic curves for different models to detect diabetic retinopathy

Metrics	Positive tear glucose	Duration of diabetes	HbA1c	Combined model
Sensitivity (%)	77.40	69.80	60.40	54.70
Specificity (%)	53.30	64.50	71.00	87.90
AUC	0.65	0.68	0.63	0.76
95%CI	0.57–0.74	0.59–0.77	0.53–0.73	0.69–0.84
p	< 0.01*	< 0.01*	< 0.01*	< 0.01*
Youden index	0.31	0.34	0.31	0.43

CI: Confidence interval

HbA1c: glycosylated hemoglobin; AUC: area under the curve

The combined model integrated tear glucose, the duration of diabetes and HbA1

^*^Statistically significant

## Discussion

The results of this cross-sectional study indicated that TG was associated with the presence and severity of DR. Positive TG suggested the presence of DR, with an increasing level of TG indicating a more advanced stage DR. TG could probably act as a convenient parameter for detecting the status of DR among patients with diabetes.

TG was first studied early in the 1930s [19]. The correlation between TG and BG concentration had been investigated using the amperometric sensors [20]. A significant correlation between TG and BG had been identified both in humans and animal models by using a wireless smart contact lens [11]. Since 1937, TG concentrations in diabetes patients have been extensively investigated [21]. An observational study confirmed that there was a proportional correlation between BG and TG in 20 individuals with diabetes [21]. Our findings were consistent with the results of previous reports. Kownacka, et al. performed the first clinical trial including 6 patients with type 1 diabetes and demonstrated that a non-blood-derived biofluid-based sensor coated with a polymer matrix can be used to accurately record tear glucose levels, which showed the glucose values from tears had an potentially acceptable ability for the management of diabetes [22]. The findings of our study initially suggested that TG was associated with DR. Confirmed with prior finding, younger age, the longer duration of diabetes and higher HbA1c were also associated with the presence of DR. A review performed using data from population-based studies globally highlighted prevalence rates of DR were increased with the duration of diabetes, values for HbA1c and blood pressure [23]. Studies of DR have consistently shown higher HbA1c was often associated with retinopathy [24]. A cohort study including 1791 participants suggested risk of progression of DR increased by 30% for each 1% increase in baseline HbA1c [25]. Diabetes duration has also been frequently investigated [24]. A cohort study recruiting 1058 patients found that the duration of diabetes was longer in the DR group than in the normal group [26]. A retrospective chart analysis of consecutive 654 patients with DR concluded that people with youth-onset diabetes mellitus were likely to present with more advanced stage of DR [27]. Previous articles suggested that establishing tear biomarkers as tools was reliable in the screening and monitoring of DR^28^. In our study, we explored whether TG could be functioned as a biomarker for DR. Further, the ability of three associates with DR including TG, the duration of diabetes and HbA1c to detect DR were compared. TG showed acceptable ability in detecting DR, and the model combined 3 factors had highest discriminative ability. However, the duration of diabetes is an unmodifiable factor, and measuring HbA1c is invasive. TG has advantage of accessibility, convenience, non-invasiveness and less cost.

The test of TG provides a convenient and noninvasive detection method for the self-management of patients with DM to prevent DR. It has a great potential to be applied to the self-monitoring of BG and the detection of DR. It is recommended that patients should seek help from eye doctors when having positive TG, especially for patients with longer duration of diabetes and higher HbA1c. This method provides a useful reference for ophthalmologists to detect DR in time and take timely interventions to delay the progression of DR.

To our knowledge, this is the first exploration of potential association between TG and DR. The main drawback of the study was the cross-sectional design, which precluded the longitudinal observation, and did not establish a causation. All the patients were recruited from a single hospital. We will continue to seek such datasets from other medical centers and a wider population for future validation.

## Conclusions

In conclusion, TG level was associated with the presence and the severity of DR. TG might become an easy-to-use, non-invasive parameter to the screening and monitoring of DR. TG testing may offer clinicians some general guidance and increase the early screening rate of DR among the high-risk diabetic population in future. A large multicenter study with a long-term follow-up is needed to fully validate findings from this study.

## Supplementary Information

Additional file 1. 

Additional file 2. Figure S1**.** **A** Tear sample collection region. A-1 Contact area of under inspection outer canthus lacrimal film; A-2 pH buffer region; A-3 Glucose test region. **B** Hydrophobic barrier region. **C** Holder portion.

Additional file 3. Figure S2**.** Four standard color blocks.

## Data Availability

No datasets were generated or analysed during the current study.
