# Supplementary material for: Tear glucose is associated with the presence and severity of diabetic retinopathy
Source: Int J Retina Vitreous. 2025 Feb 6;11:13. doi: 10.1186/s40942-025-00636-x (PMC11800454; doi:10.1186/s40942-025-00636-x)
Supplement: Supplementary file 1 — Additional file 1. [file 40942_2025_636_MOESM1_ESM.docx]

**Supplementary materials**

**Detailed use of rapid tear glucose qualitative test strip**

The rapid tear glucose qualitative test strip made by filter paper has a Schirmer’s strip shape including tear sample collection region (A), hydrophobic barrier region (B) and holder portion (C). The tear sample collection region includes the leading portion contact area (A-1) with outer canthus to absorb tear from lacrimal film, pH buffer region (A-2) to neutralize the tear sample and the last portion is the glucose test region (A-3) to analysis the tear glucose concentration by glucose oxidase coupled with Trinder’s reaction.

First, the contact area of the tear sample collection region of the strip absorbs tear sample from lateral canthus by principle of capillary after setting the test strip within the under inspection eye outer canthus, and then the tear will go up through the pH buffer region neutralizing the sample and then arriving at the he glucose test region, the tear glucose concentration is analyzed by glucose oxidase coupled with Trinder’s reaction, the chromogen will be developed if the tear glucose concentration in excess of 0.153mmol/L, the up tear will be blocked by hydrophobic barrier region and could not cross the barrier so within the tear sample collection region of the strip will absorb the same volume tear from the tear film of the each patient to evaluate the tear glucose concentration (Figure S1).

**The production of standard color blocks**

Dropping 3μl each of the four glucose standards (S1 0.153mmol/L, S2 0.306mmol/L, S3 0.613mmol/L and S4 1.226mmol/L) to the tear sample collection region of the rapid tear glucose qualitative test strip. After the chromogen development completed, the result of the four standards concentration as the standard color blocks will be printed on the technical manual (Figure S2).
