# Supplementary figures and images for: Tear glucose is associated with the presence and severity of diabetic retinopathy
Source: Int J Retina Vitreous. 2025 Feb 6;11:13. doi: 10.1186/s40942-025-00636-x (PMC11800454; doi:10.1186/s40942-025-00636-x)

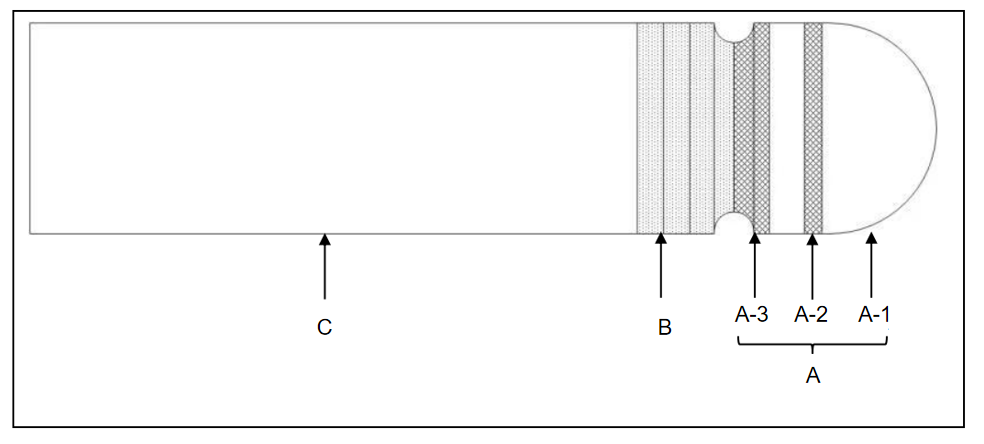

Supplement: Supplementary file 2 — Additional file 2. Figure S1. A Tear sample collection region. A-1 Contact area of under inspection outer canthus lacrimal film; A-2 pH buffer region; A-3 Glucose test region. B Hydrophobic barrier region. C Holder portion. [file 40942_2025_636_MOESM2_ESM.tif]

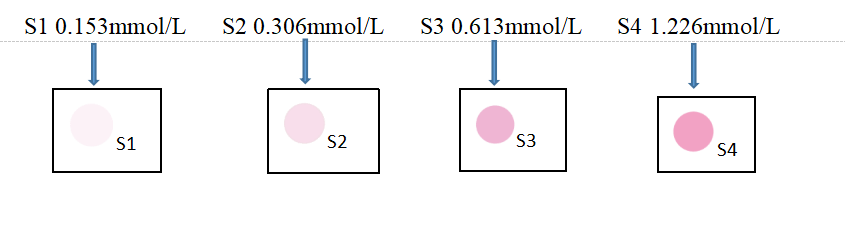

Supplement: Supplementary file 3 — Additional file 3. Figure S2. Four standard color blocks. [file 40942_2025_636_MOESM3_ESM.tif]
